# NOSE-OE: A molecular signaling map of the olfactory epithelium

**DOI:** 10.1016/j.isci.2026.117818

**Published:** 2026-10-15

**Authors:** Shailendra Gupta, Federica Genovese, Krishna P. Singh, Alessandro Nicoli, Francesco Ferri, Malgorzata M. Kogut-Günthel, Dominique Fastus, Suchi Smita Gupta, Matti Van Welzen, Olaf Wolkenhauer, Antonella Di Pizio

**Affiliations:** 1 Department of Systems Biology & Bioinformatics, University of Rostock, Rostock, Germany; 2 Department of Clinical Neurosciences, University of Geneva, Geneva, Switzerland; 3 Unité Biologie Fonctionnelle & Adaptative, Université Paris Cité, Paris, France; 4 Monell Chemical Senses Center, Philadelphia, PA, USA; 5 Clinic and Policlinic for Dermatology, Venereology, and Allergology, University Medical Center Rostock, Rostock, Germany; 6 Leibniz Institute for Food Systems Biology at the Technical University of Munich, Freising, Germany; 7 Professorship for Chemoinformatics and Protein Modeling, Technical University of Munich, Freising, Germany; 8 Structural Biology and NMR Laboratory, Linderstrøm-Lang Centre for Protein Science, Department of Biology, University of Copenhagen, Copenhagen, Denmark; 9 Atomistic Modeling Center, Munich Data Science Institute, Technical University of Munich, Garching, Germany

**Keywords:** systems biology, chemical senses, olfaction, cell, molecular interactions

## Abstract

The olfactory epithelium (OE) is a dynamic and complex sensory tissue in which continuous regeneration, neuronal differentiation, and tissue maintenance depend on coordinated molecular and cellular signaling. We developed NOSE-OE (Network of Signaling Events in the Olfactory Epithelium), an open-access knowledge resource that integrates mechanistic evidence from more than 300 publications into a standardized, computer-readable signaling network. NOSE-OE comprises over 500 molecular elements and 1,000 curated interactions and is implemented within the MINERVA platform for interactive exploration and network analysis. By connecting fragmented experimental evidence within a unified molecular framework, NOSE-OE enables the systematic investigation of signaling processes governing OE homeostasis and responses to perturbation. The resource provides a foundation for hypothesis generation and systems-level investigation of olfactory epithelial biology across physiological and disease contexts.

## Highlights

• NOSE-OE connects over 500 molecular elements through 1,000 curated interactions
• Mechanistic knowledge is standardized into a computer-readable molecular signaling map
• NOSE-OE enables systems-level exploration of olfactory epithelium biology

## Graphical abstract

**Figure:**
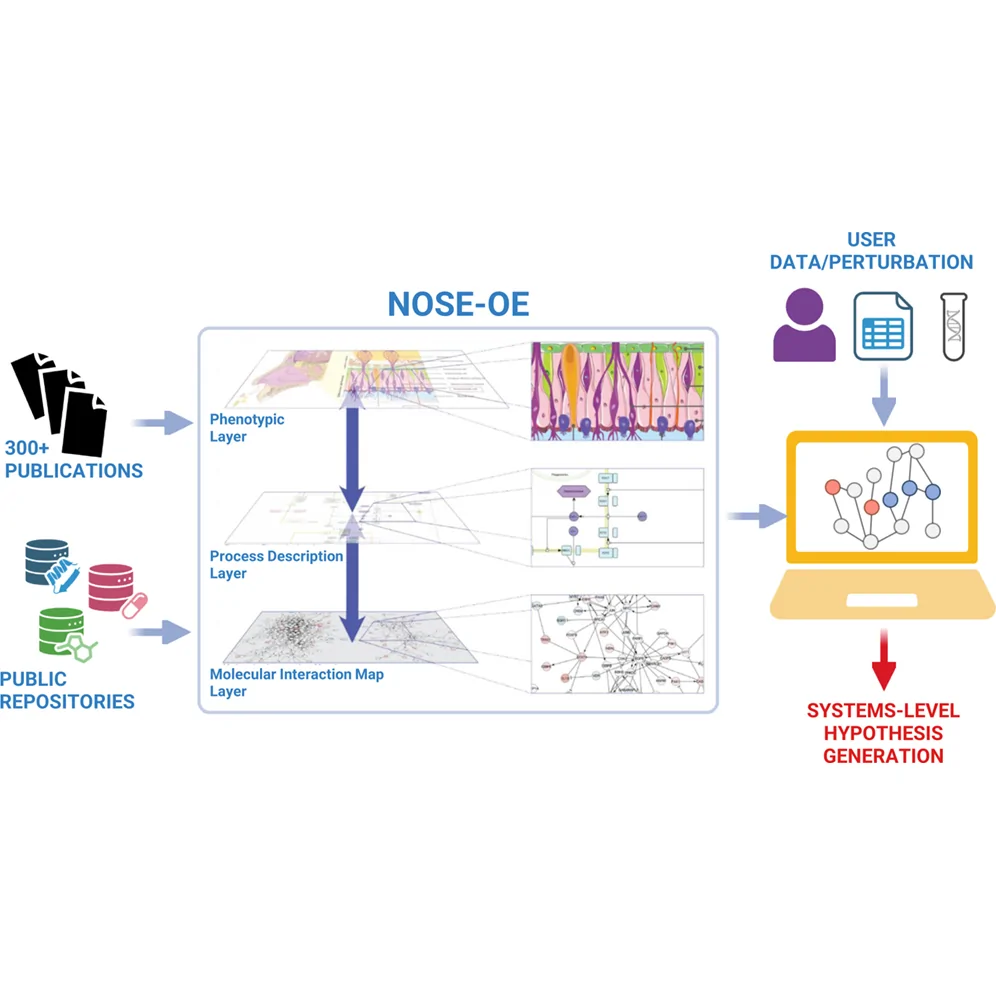

## Teaser

Biological sciences; Molecular network; Sensory neuroscience

## Introduction

Olfaction is responsible for the detection of volatile molecules, odors, present in the surrounding environment, allowing for fundamental survival behaviors, such as identifying food sources, finding mates, and avoiding predators. The detection of odors takes place in the olfactory epithelium (OE), a specialized epithelium located in the olfactory region of the nasal cavity, mainly along the superior nasal concha and the adjacent upper part of the nasal septum, near the cribriform plate.^1^^,^^2^ The OE is organized as a pseudostratified epithelium (Figure 1), and at its functional core reside the olfactory sensory neurons (OSNs), which are bipolar neurons possessing a single, unbranched dendrite that extends toward the apical surface of the OE. Their dendrite terminates in an expanded dendritic knob from which multiple cilia emerge and project into the nasal lumen. The olfactory cilia contain the molecular components essential for odorant signal transduction, including olfactory receptors that are directly exposed to the external environment, enabling the initial detection of odorant molecules. Olfactory receptors are the largest subfamily of G protein-coupled receptors (GPCRs), and their number varies across species, approximately, 1,000 functional genes in mice (Olfrs) and about 400 genes in humans (ORs). Each mature OSN expresses a single OR allele (or can also co-express an OR pseudogene).^3^^,^^4^ Axons from OSNs expressing same OR converge onto a small, stereotyped set of glomeruli (typically one medial-lateral pair per bulb), creating a spatially organized odor map.^5^^,^^6^^,^^7^ Immature OSNs (iOSNs), however, exhibit low-level OR expression and initiate synaptic signaling even during differentiation.^8^^,^^9^^,^^10^^,^^11^ The OE undergoes constant renewal sustained by basal stem cells, globose basal cells (GBCs) and horizontal basal cells (HBCs), which continuously regenerate OSNs and other OE cell types throughout life.^12^^,^^13^^,^^14^^,^^15^^,^^16^

**Figure 1:**
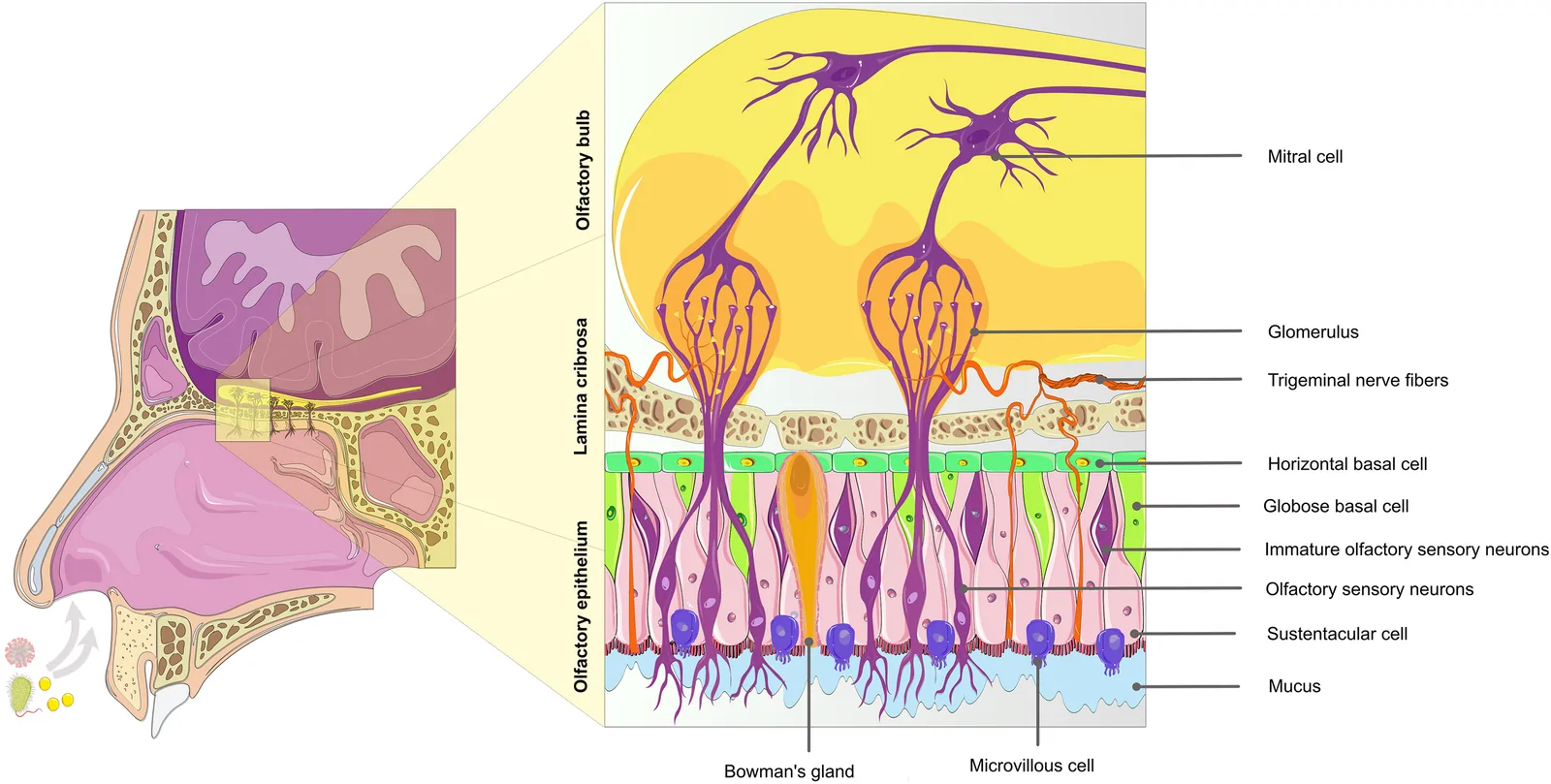
Phenotypic representation of olfactory epithelium cell types and compartments The olfactory epithelium is a pseudostratified tissue composed of primary sensory neurons (olfactory sensory neurons [OSNs]), supporting cells (sustentacular [Sus] cells, and microvillous cells [MVCs]), and two basal stem cell populations (horizontal basal cells [HBCs] and globose basal cells [GBCs]) essential for regeneration. Specialized structures such as Bowman’s glands and the mucous layer provide functional support and protection. OSN axons form the olfactory nerve and project centrally to the olfactory bulb (OB), where axons expressing the same odorant receptor (OR) converge onto dedicated glomeruli and synapse with mitral cells, the main projection neurons of the OB. The cellular compartments localized in the OE, and their interactions form the structural basis for the NOSE-OE molecular maps.

GBCs comprise 2% of the OE cells and actively proliferate to regenerate all cell types of the OE.^9^^,^^17^^,^^18^^,^^19^ In contrast, HBCs serve as reserve stem cells, which are activated only after severe injury.^13^^,^^20^^,^^21^ Proliferating HBCs have the function to replenish the gradually depleting GBCs.^22^^,^^23^

Sustentacular (Sus) cells are situated in the most apical region of the OE.^24^^,^^25^ These glia-like cells regulate ionic balance, metabolize toxins, and secrete mucus components, such as lysozymes and odorant-binding proteins.^26^^,^^27^^,^^28^^,^^29^ Alongside the Sus cells are Bowman’s glands, also known as olfactory glands,^30^ which consist of an acinus in the lamina propria and a secretory duct opening onto the OE surface. Bowman’s glands play a key role in maintaining the mucosal layer that coats the epithelial surface.^30^^,^^31^ The mucous layer is essential to ensure a functional olfactory transduction cascade as it mediates odorant delivery to the ORs and provides the proper concentration of ions for signal transduction. The mucous layer also has an essential role in protecting the OE from toxicants and pollutants.^32^ Finally, microvillous cells (MVCs) further shield the OE from bacterial and chemical hazards,^33^^,^^34^^,^^35^^,^^36^^,^^37^ underscoring the tissue’s multilayered defense system.

OSNs are not the only sensory cells located in the OE. The ophthalmic division of the trigeminal nerve contains sensory endings that innervate the OE and enable the detection of inhaled irritants.^38^ Two different populations of trigeminal sensory fibers can be distinguished: peptidergic and non-peptidergic.

The cell bodies of these neurons are located in the trigeminal ganglion outside the OE. Non-peptidergic fibers have only sensory functions, whereas peptidergic fibers, upon stimulation, can also release various proinflammatory neuropeptides within the OE, thereby initiating protective inflammatory responses.^39^

Together, these diverse cell types form a tightly coordinated, self-renewing system that preserves a functional OE despite the constant environmental challenge. External threats (pathogens, pollutants) and internal stressors (aging, neurodegeneration, inflammation) disrupt critical pathways for OE homeostasis, ranging from mucus secretion to stem cell differentiation. However, while individual molecular mechanisms have been extensively studied, a systems-level mechanistic understanding of how signaling pathways integrate across OE cell types remains elusive.

Here, we introduce NOSE-OE (Network of Signaling Events in the Olfactory Epithelium), the first compartment-resolved, SBML/SBGN-encoded molecular map that unifies odor transduction, epithelial regeneration, and inflammatory signaling across all major OE cell types (Figure 1). Leveraging the Disease Map approach,^40^^,^^41^^,^^42^^,^^43^ NOSE-OE combines literature-curated interactions with computational modeling in an interactive platform powered by MINERVA,^44^ enabling systematic exploration of OE regulatory networks.

## Results and discussion

### Knowledge extraction and standardization of OE molecular components

Disease maps support the exploration of state-of-the-art knowledge about a biological system or disease context.^42^^,^^43^^,^^45^ These comprehensive, accessible resources collect validated knowledge about biological processes, molecular mechanisms, phenotypes, and their interactions. Encoding this knowledge in a standardized format enables the use of established analytical tools to extract insights from complex interactions, facilitate data integration, and perform *in silico* experiments for advanced visualization and analysis.

This methodology has been successfully applied to numerous complex biological systems.^43^^,^^46^^,^^47^ Here, we extend this paradigm to the OE, generating the NOSE-OE molecular map, a unified compartment-resolved representation of its cell-to-cell communication and regulatory networks. Our approach began with extensive manual curation and literature mining of over 300 peer-reviewed publications and public repositories, focusing on identifying and validating molecular elements, including receptors, ligands, enzymes, transcription factors, and small molecules, essential for OE function. This process yielded a curated collection of more than 500 molecular elements and over 400 specific molecular interactions, forming one of the most detailed mechanistic representations of the OE to date.

This validated molecular information was then integrated and visualized using the CellDesigner platform, adhering to standard Systems Biology Graphical Notation (SBGN) guidelines to ensure clarity and interoperability. Figure 2 provides a high-level conceptual overview of submap connectivity and serves as a legend for the SBGN symbols used throughout this work.

**Figure 2:**
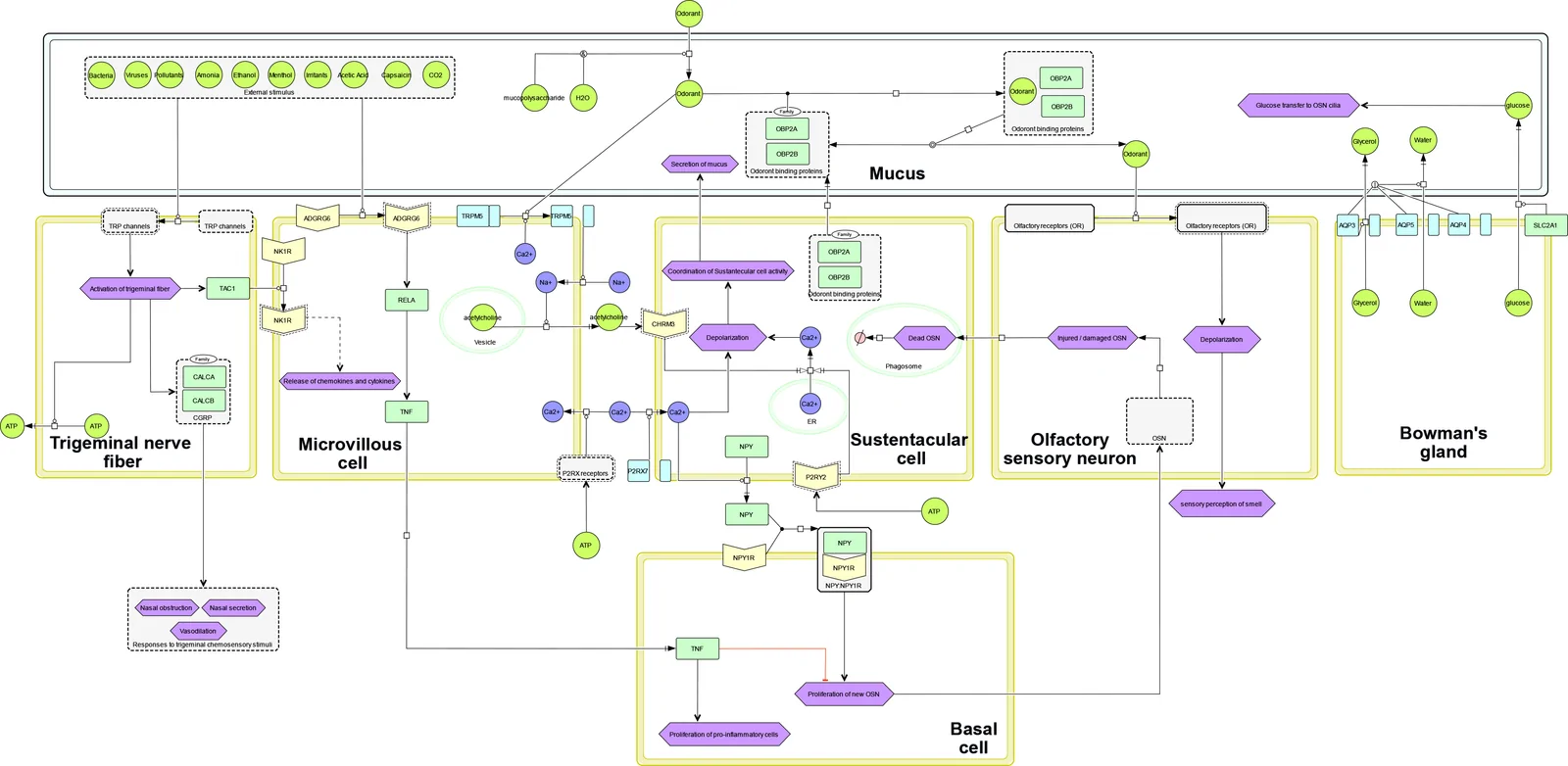
NOSE-OE molecular map Molecular interactions involved in the regulation of various phenotypes (purple), such as “secretion of mucus” or “olfactory signal,” are represented using Systems Biology Markup Language (SBML) and Systems Biology Graphical Notation (SBGN). The advantage of such standardized representations is that they can be analyzed using bioinformatics and systems biology approaches, including graph-theoretical analyses of the topological structure of large networks, statistical analyses, logical and mechanistic modeling, and simulation. The eight submaps correspond to OSNs, sustentacular and microvillous cells, globose and horizontal basal cells, Bowman’s glands, mucous layer, and trigeminal fibers, each with Gene Ontology-aligned phenotype nodes for downstream enrichment and perturbation analysis. Note: individual network elements and fine-grained details are scaled for layout visualization and are not intended for direct reading from this static schematic; full individual elements, submaps, and complete network topologies are fully accessible and interactive on the web platform.

Eight distinct maps were designed to reflect the physiological architecture of the OE: these include specialized cell types (OSNs, Sus cells, microvillous cells, GBCs, and HBCs), support organs (Bowman’s gland), the complex extracellular matrix (mucus), and associated nervous input (trigeminal nerve fiber). These maps allow users to interrogate not just the presence of a molecule, but its full context: its location within a specific cell type, its role in defined signaling processes, and its position within the broader intercellular signaling landscape of the OE. In the following subsections (Boxes 1, 2, 3, 4, 5, 6, 7, and 8), we detail the structure, key molecular elements, and functional insights derived from each of these individual maps.

Box 1 **OSN map**

*Direct MINERVA Interactive Model: NOSE-OE OSN Submap*

The OSN map is built as a unique compartment.

• The top region contains the cilia with the transduction cascade, including olfactory receptors, adenylate cyclase 3 (ADCY3), cyclic nucleotide-gated ion channels (CNGs), and anoctamin 2 (ANO2), as well as the components necessary to terminate the odor signal, like the phosphodiesterase 1 C (PDE1C) and the K^+^-dependent Na^+^/Ca^2+^ exchanger 4 (NCKX4).^48^
• Moving down toward the cell body, we find soma components. At the soma and axon hillock, we represented the voltage-gated Na^+^ (Nav1.7) and Ca^2+^ (Cav1.3) ion channels necessary to generate the action potential and the Ca^2+^-activated K channel (KCNN1) required to modulate the action potential firing pattern.^49^^,^^50^^,^^51^ Purinergic receptors P2RY2 and P2RX4, which have been shown to affect the OSN excitability,^52^^,^^53^^,^^54^ are also included.
• All the elements located in the glomerular synapsis are located at the bottom of the cell compartment. The olfactory signal generated by the OSNs converges at the glomeruli in the OB. Each glomerulus includes the axon terminals of OSNs expressing the same OR. These axons make synaptic contacts with the mitral/tufted (M/T) cells, the main output of the OB.^55^ Within the glomeruli, periglomerular interneurons regulate the signal delivered to the M/T cells.^56^^,^^57^ Periglomerular interneurons and M/T cells are activated by glutamate released by the OSN pre-synaptic terminals.^58^ The activation of the periglomerular neurons causes feedback inhibition onto the OSN terminals mediated by the release of dopamine and GABA. Our map does not include the full complexity of the interglomerular network, but rather a simplification of both GABAergic and dopaminergic feedback from periglomerular cells to the OSN terminals and M/T cells.

Box 2 **HBC map**

*Direct MINERVA Interactive Model: NOSE-OE HBC Submap*

• Expression levels of the transcription factor p63 (ΔN isoform) function as a master switch that controls the transition of HBCs between activation and dormancy.^13^^,^^59^ It was demonstrated that the downregulation of p63 is both necessary and sufficient to achieve the activation of HBCs.^23^^,^^60^
• Notch signaling appears to play a role in regulating the level of p63 expression.^61^ Notch1 and Notch2, their ligands, as well as Hes1, the usual downstream transcription factor mediator of Notch action, are expressed by HBCs.^61^ Genetic excision of Notch1 enhances the low rate of spontaneous HBC activation in the uninjured OE. In contrast, overexpression of the Notch1 intracellular domain elevates the downstream factor Hes1 and increases p63 expression.^13^^,^^60^
• Canonical *Notch* signaling determines the balance between neurons and non-neuronal cells during the restoration of the epithelium after injury, with RBPJ, the canonical Notch transcriptional cofactor, exhibiting haploinsufficiency in setting the balance among the regenerating populations.^62^ However, as p63 is expressed in both quiescent and proliferating HBCs, it cooperates with other factors in the cell to regulate the transition from quiescence to proliferation. Recent genetic studies have identified Wnt signaling, Yap signaling, and inflammatory signaling as additional contributors to OE regeneration after injury.^63^^,^^64^^,^^65^^,^^66^^,^^67^ However, given the current uncertainty regarding the underlying mechanisms, these pathways were not incorporated in the current version of NOSE-OE maps.

Box 3 **GBC map**

*Direct MINERVA Interactive Model: NOSE-OE GBC Submap*

• The GBCs are a heterogeneous population of cell progenitors in the olfactory epithelium. Some GBCs are committed to the OSN lineage and support normal neuronal turnover in the olfactory epithelium, by passing through neuronal precursor cells (NPCs); they give rise to immature OSNs (iOSNs), which then differentiate into mature OSNs (mOSNs).^13^^,^^68^^,^^69^^,^^70^^,^^71^ Others are suggested to act as multipotent progenitors to generate all the other types of OE cells.^72^^,^^73^^,^^74^^,^^75^ GBCs also differ in their proliferative behavior: some cycle rapidly but have limited total expansion capacity, while others are label retaining and slowly dividing.^19^
• The regulation of adult neurogenesis and regeneration in the OE involves a network of transcription factors, and the complete regulatory landscape remains incompletely understood.^76^ Sox2 and Pax6, expressed in Sus cells, GBCs, NPCs, and HBCs, may play multiple and complex roles in the regulation of olfactory neurogenesis as well as in the maintenance of multipotency.^77^ Ascl1 (also known as Mash1) and Hes1 control cell fate decision in the OE, influencing both neuronal (OSN) and non-neuronal lineages (especially the supporting cells). Ascl1 and Hes1 act as transcriptional activators or suppressors for each other. While Ascl1 promotes neuronal lineage, Hes1 represses it.^72^ Hes1 has important roles in non-neuronal differentiation in the OE.^78^ Ngn1 and NeuroD act as downstream of Ascl1 and are expressed in GBCs and INPs (late-stage neuronal precursor cells). Hes5 functions as a repressor in neurogenesis synergized with Hes1, as it regulates the expression of Ngn1 exclusively.^78^^,^^79^

Box 4 **Trigeminal peptidergic fiber map**

*Direct MINERVA Interactive Model: NOSE-OE Trigeminal Submap*

• The trigeminal nerve detects various stimuli, including pollutants and irritants like capsaicin, CO_2_, and ethanol, using transient receptor potential (TRP) channels, such as TRPA1 and TRPV1.^80^ TRPA1 and TRPV1 are non-selective cation channels activated by thermal and chemical stimuli and are often co-expressed on trigeminal peptidergic fibers. Trigeminal peptidergic fibers also express ionotropic (P2X) and metabotropic (P2Y) purinergic receptors, which also contribute to trigeminal fiber activation in the presence of ATP.^81^^,^^82^
• Fiber activation induces additional ATP release, which can increase intracellular Ca^2+^ in Sus cells through the activation of P2X7 and P2Y2 receptors. Pro-inflammatory neuropeptides, such as substance P ([SP], encoded by TAC1) and calcitonin gene-related peptide (CGRP), are also released by these nociceptive trigeminal fibers.^82^^,^^83^
• Released CGRP modulates OSN odor responses and induces nasal vasodilation, obstruction, and mucus secretion,^84^^,^^85^^,^^86^ while SP activates the MAPK signaling cascade in MVCs to drive the release of chemokines and cytokines.^35^

Box 5 **Microvillous cell map**

Direct MINERVA Interactive Model: NOSE-OE MVC Submap

• Microvillous cells can be classified into distinct subpopulations based on their morphology, cell marker expression, and functional properties.^37^^,^^87^^,^^88^^,^^89^^,^^90^^,^^91^ These include IP3R3+/BSND+ cells (also called ionocytes) and TRPM5-positive cells (also called nasal tuft cells).^92^
• Ionocytes are the predominant source of neuropeptide Y (NPY) in the OE. NPY release is triggered by increases in intracellular Ca^2+^, mediated through the IP3 receptor subtype 3, and is upregulated following epithelial injury.^93^^,^^94^ NPY release from this population supports progenitor cell proliferation and contributes to tissue homeostasis and regeneration after injury.^95^
• Nasal tuft cells (TRPM5-positive cells) are the ones represented in NOSE-OE. They are a likely source of acetylcholine (Ach) in the OE, and its release is finely regulated by increases in intracellular Ca^2+^ levels.^90^^,^^96^^,^^97^ Intracellular Ca^2+^ increase can be triggered through different mechanisms, such as the activation of P2X receptors by ATP, which leads to Ca^2+^ influx (calcium influx from the extracellular space),^90^^,^^96^^,^^97^ or through GPCR-mediated signaling pathways that mobilize intracellular Ca^2+^ stores, as in the case of activation of the sweet taste receptor, expressed on this cell’s membrane.^98^ TRPM5 ion channels can further amplify Ca^2+^ signals, ultimately promoting Ach release from vesicles.^34^^,^^96^ These cells respond to external pathogens through activation of adhesion G protein-coupled receptor G6 (ADGRG6), followed by secretion of TNFα via NF-κB signaling pathway. TNFα signaling inhibits the neuroregenerative phenotype of the HBCs and activates cytokine-mediated immune defense pathways within the OE.^99^ These mechanisms are represented on the laterals and apical membranes of the MVC map.

Box 6 **Sustentacular cell map**

*Direct MINERVA Interactive Model: NOSE-OE Sus Submap*

• Sus cells play an important role in supporting and isolating the OSNs while maintaining the OE microenvironment.^100^^,^^101^ The different pathways that contribute to these different functions are included in the Sus cell map.
• In the apical region of the map, we included biotransformation enzymes, lysozymes, xenobiotic-metabolizing enzymes, and OBPs as well as AQP3 and AQP5, which are expressed on the apical membrane of Sus cells.^102^^,^^103^
• Sus cells maintain tissue integrity by phagocytosing dead neurons and driving mucus secretion,^104^^,^^105^^,^^106^ coordinated by acetylcholine internalized by muscarinic M3 receptors (represented on the basal membrane in the map).^34^^,^^107^
• Sus cells produce and respond to several neurotrophic and neuromodulatory molecules, such as acetylcholine, NPY and ATP, endocannabinoids, and insulin, to enable communication across OE cell types.^108^^,^^109^^,^^110^^,^^111^
• Purinergic P2X/P2Y receptor activation triggers increase of intracellular Ca^2+^.^82^^,^^112^^,^^113^ Secretion, proliferation, and development in Sus cells are regulated by Ca^2+^ signaling, and via Ca^2+^-dependent exocytosis, Sus cells may release chemical signals to basal cells, neuronal precursors, or neurons. Ca^2+^ fluxes may act as signal for cell turnover, as Ca^2+^ oscillations and waves in glial cells are known to regulate cell turnover and neurogenesis.^110^^,^^114^ Intracellular Ca^2+^ can drive production and release of NPY,^108^^,^^115^^,^^116^ a neurotrophic factor responsible for the proliferation of basal cells. Sus cells also have functional Ca^2+^-activated Cl^−^ channels (ANO1 channels) producing Cl^−^ efflux, which contributes to membrane depolarization and mucus secretion dynamics, thereby supporting Cl^−^ homeostasis.^113^ Cytoplasmic Cl^−^ concentration is also necessary for endocytic trafficking, fundamental in the Sus cells for their detoxification after the metabolization of xenobiotics in the intracellular compartments.^117^^,^^118^^,^^119^

Box 7 **Bowman’s glands map**

*Direct MINERVA Interactive Model: NOSE-OE Bowman Submap*

• Bowman’s glands, which are dedicated to producing major mucus constituents, including defense proteins, glucose, water, and ions, are composed of two different cell types that are represented as separate compartments on the map, i.e., the acinar and the duct cells.
• Water is secreted from the Bowman’s glands through aquaporins (AQPs) 3, 4, and 5.^31^^,^^102^ AQPs are membrane channel proteins that allow the permeation of water and in some cases, small solutes across cellular membranes.^120^ In Bowman’s glands, we have AQP4 and AQP5 that are permeable to water only, while aquaglyceroporin AQP3 is permeable to water as well as glycerol, urea, and small solutes.^121^ AQP3 and AQP5 were localized in the apical membrane in the secretory acinar cells, whereas AQP4 is localized on the membrane of duct cells.
• To maintain mucosal hydration and osmotic balance, acinar cells release chloride ions into the mucus via ANO1 channels,^122^ alongside glucose transported by SLC2A1, which is subsequently internalized by OSN cilia via the SLC2A3 transporter.

Box 8 **Mucus layer map**

Direct MINERVA Interactive Model: NOSE-OE Mucus Submap

• Composed primarily of water, inorganic salts, and viscoelastic glycoproteins,^123^ the mucus map includes solubilized odorants alone or bound to odorant-binding proteins (OBP2A and OBP2B).^124^ OBPs are secreted by Sus cells and facilitate interactions between odorant molecules and ORs.^103^ After the odorant is released, OBPs are either enzymatically degraded or recycled via transport back to the Sus cells.^125^
• The map integrates additional transport proteins, including lipocalins and bactericidal/permeability-increasing (BPI) fold proteins.^126^^,^^127^
• The mucus also acts as the first line of defense, thanks to proteins such as pathogen-trapping glycoproteins,^31^ lysozyme-degrading bacterial cell walls,^128^ antimicrobial BPIFA1 (previously known as PLUNC),^129^ and xenobiotic-clearing biotransformation enzymes, including cytochrome P450s and UDP glucuronosyltransferases.^130^^,^^131^^,^^132^

### Intercellular interaction networks in the OE

Beyond mapping individual cellular components, the core strength of the NOSE-OE map is the explicit representation of cross-compartmental communication. These interactions, which include signaling between distinct cell types, molecular transport across compartments, and shared regulatory mechanisms, are essential for understanding how processes across the OE are functionally integrated.

Structural analysis of the interactome (Figure 3) illustrates the complexity and density of the OE’s regulatory landscape by plotting direct protein-protein interactions (PPIs) established across the NOSE-OE submaps. To provide a tractable overview of connectivity, compartmental localization is encoded by color-coded contours surrounding each node. Proteins involved in cross-compartmental signaling or shared regulatory functions are distinguished by multiple concentric rings.

**Figure 3:**
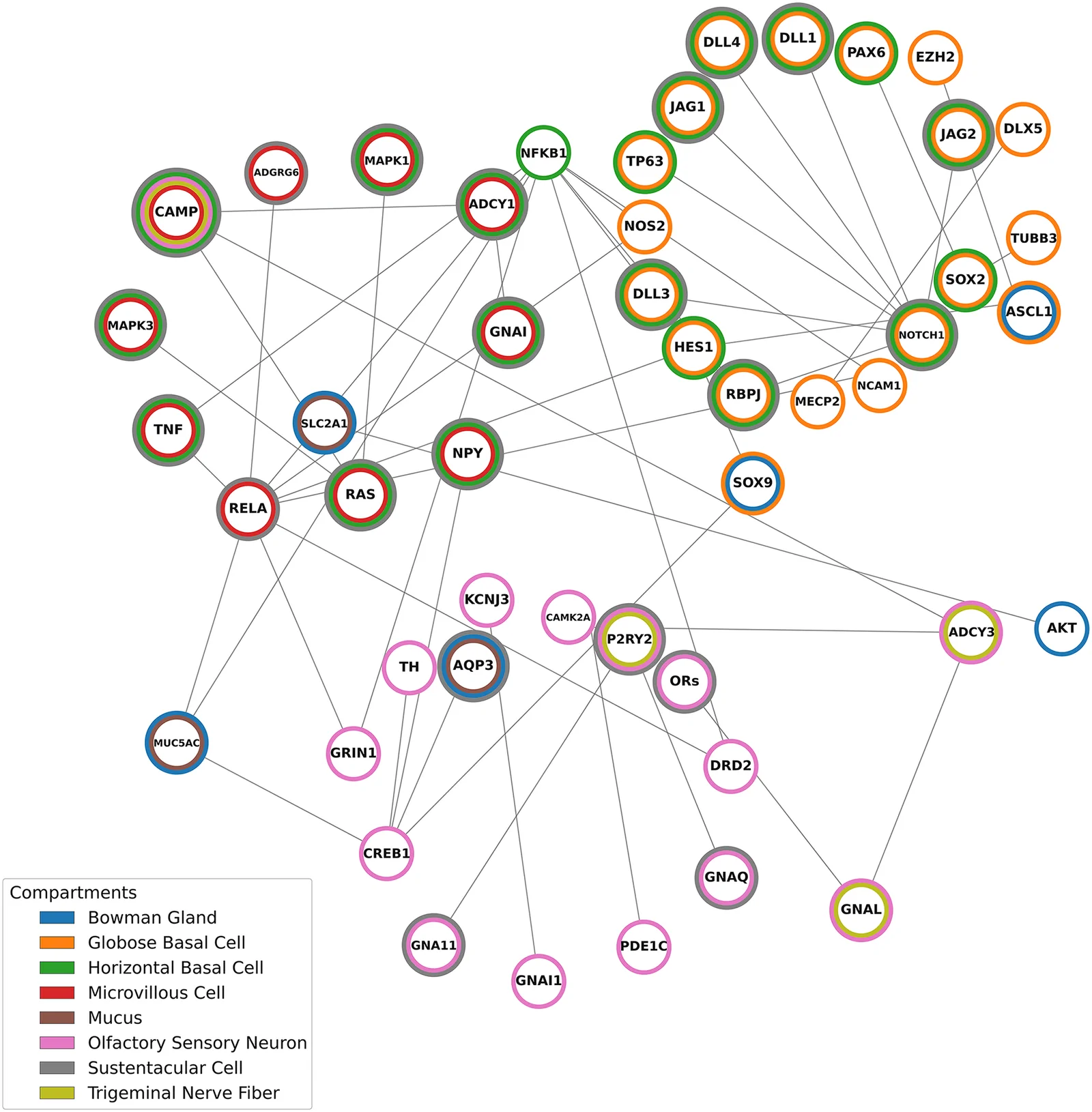
PPI subnetwork derived from the NOSE-OE map The network displays all direct PPIs between protein entities only curated across the eight NOSE-OE submaps. Nodes represent proteins annotated with HGNC gene symbols, and edges represent experimentally supported interactions. Node contour colors encode compartmental localization, highlighting whether a protein participates in signaling within neuronal, non-neuronal, extracellular, or progenitor compartments, while multiple concentric rings indicate proteins shared across several compartments. Phenotype nodes and small molecules are not shown in this PPI projection and are instead represented in the underlying CellDesigner/MINERVA maps.

This signaling convergence is most evident in high-degree hub nodes such as cAMP, which features multiple layers of color, visually representing the involvement in neuronal, non-neuronal, and progenitor compartments. A key feature of this network configuration is its high degree of shared protein involvement and signaling convergence, where numerous critical regulatory components participate in interactions spanning multiple distinct cell compartments. This structural arrangement underscores the structural integration of the OE model, suggesting a framework where multiple molecular components enable coordinated responses to environmental and physiological perturbations.

Topologically, the ubiquity of gray contours throughout the interactome highlights the structural connectivity associated with Sus cells. When comparing the relative connectivity profiles across the distinct cell compartments, nodes associated with Sus cells exhibit a high concentration of cross-compartmental edges that directly interface with multiple network layers. Within the boundaries of this simplified network layout, their dense connection profile positions them as a structural “connective tissue,” acting as an architectural integrative hub for the modeled OE. Though traditionally categorized primarily as structural glial support cells, this network-level connection profile suggests that they are architecturally optimized to serve as key intermediaries, routing potential signals from the mucous layer down to the basal layer. It is critical to note that the presence of these shared connections and compartmental annotations does not inherently imply that sustentacular cells exert functional dominance. However, this centralized architectural configuration aligns with empirical observations where COVID-19 sensory loss results from primary support cell infection rather than direct neuronal damage.^133^^,^^134^ This reinforcement supports a more holistic, systems-level perspective on chemosensory tissues. Whereas previous efforts have largely mapped the molecular mechanisms of OSNs, the surrounding non-neuronal components appear structurally essential to the network architecture required to maintain and regulate neuronal functions.

The interactome further reveals how these “sensing” compartments, Sus cells and MVCs, are positioned to act as regulatory switchboard. The ADGRG6-RELA interaction^99^ illustrates this, providing a localized case study of cross-compartmental connectivity.

The adhesion GPCR ADGRG6 (GPR126) likely provides a mechanism for a cell to sense mechanical or environmental changes at its surface, which then maps directly into the core inflammatory and cell-fate pathway governed by the transcription factor RELA (NF-κB p65).^135^^,^^136^ While the ADGRG6-RELA axis is well characterized in the context of Schwann cell regeneration following peripheral nerve injury, its role in the olfactory system is less documented. Our model identifies this interaction as a potential molecular bridge between apical sensing and basal response. In this structural pathway, activation of the ADGRG6-RELA axis results in TNFα production, which is mapped to recruit and activate macrophages. Crucially, TNFα also induces a functional shift in HBCs, switching their phenotype from neuroregeneration to immune defense (dashed pathway, Figure 3).^99^^,^^136^ This interaction demonstrates how the interactome captures the directional “instructions” sent from the surface-sensing support cells to the basal regenerative niche, providing an accessible reference model to guide future dynamic validation studies.

This analysis shows that multiple proteins participate in signaling interactions spanning neuronal, non-neuronal, and extracellular compartments, enabling coordinated responses to environmental and physiological perturbations. However, it is important to note that the network presented in Figure 3 represents a static simplification of the molecular network, where proteins are the only elements mapped. This strategic abstraction was chosen intentionally to provide a tractable, legible blueprint of primary cross-compartmental axes without overwhelming the reader with the complexity of the interactome. The actual molecular topology is far denser, underscoring the necessity of a structured, interactive, digital platform to navigate and functionally analyze the nonlinear, multifaceted molecular interactions that govern OE function. To meet this need, the complete, non-attenuated molecular topology, including detailed metabolic cascades, non-protein elements, and nonlinear regulatory loops, is fully retained and dynamically interactive within the MINERVA platform framework.^42^

### NOSE-OE: A unified molecular framework via the MINERVA platform

To translate the curated molecular maps from static diagrams into a functionally integrated and analyzable molecular framework, we integrated all eight compartmental submaps into a single, unified system using the MINERVA platform.^43^ MINERVA serves as the primary resource portal for the NOSE-OE knowledge base, providing a web-based platform for the interactive visualization and analysis of the OE molecular network.^44^ This integration overcomes the fragmentation inherent in individual CellDesigner files, providing a single, comprehensive view of cell-to-cell communication and regulatory pathways spanning the OE’s complex layers. As a result, network-level dependencies and cross-compartmental signal propagation become directly observable. The modular architecture allows continuous expansion as new experimental evidence emerges, supporting community-driven updates and long-term sustainability. As demonstrated by the AIR,^137^ this approach enables the maps to evolve from a static knowledge repository into a living platform supporting translational research and system-level modeling.

The MINERVA platform enhances the utility of the NOSE-OE map by providing robust integrative features. All submaps are available with interactive zoom functionality, allowing users to explore key molecules and pathways that regulate specific biological processes or phenotypes shown in the overviews. For every molecular element, the MINERVA environment automatically establishes direct, context-aware hyperlinks to a wide array of external scientific databases (e.g., miRTarbase, UniProt, GenBank, PubMed, etc.). All mapped reactions are annotated with supporting evidence, which includes direct links to PubMed IDs, accelerating the processes of data validation and hypothesis generation. Biological processes are represented as phenotype nodes, defined according to the Gene Ontology resource. This evidence-centric curation ensures that the map captures mechanistic knowledge rather than statistical associations alone, distinguishing it from pathway collections derived primarily from high-throughput datasets.

By providing a unified, navigable, and integrative platform, the NOSE-OE molecular map enables access to and analysis of the complex molecular signaling that governs the early stage of olfactory processing. The NOSE-OE molecular map is designed to serve diverse research communities involved in olfaction by providing tailored entry points to access manually curated information at the cellular, pathway, and molecular levels. To make the maps accessible to a wide audience, the NOSE-OE MINERVA instance is linked to a chat interface powered by natural language models (LUMINAR: Leveraging Unified Molecular Interaction Networks for Analysis and Reasoning), allowing users to interactively explore system-level responses to molecular perturbations within the maps. Furthermore, through the MINERVA platform, we deliver a community-driven resource, allowing users to submit new literature, flag missing references, or suggest cross-species updates for future map releases. Together, this versatile analytical ecosystem establishes the NOSE-OE map as an extensible knowledge base and a dynamic platform for generating hypotheses across various physiological and pathological contexts.

### Perturbation analysis reveals functional crosstalk in the OE

The integration with LUMINAR enables exploratory *in silico* perturbation analyses and hypothesis-driven investigation of the Molecular Interaction Map by propagating changes in the activity of selected elements through the directed network topology and visualizing their effects on downstream phenotype nodes. This interactive visualization allows researchers to track how a molecular perturbation in one cell type propagates through the network to affect other compartments. By linking molecular changes to predicted phenotypic outcomes, the NOSE-OE platform provides a tool for exploring olfactory epithelial signaling and potential interventions. As an example of an application of the NOSE-OE maps, we utilized the LUMINAR plugin to perform an *in silico* perturbation analysis to investigate the downstream systemic effects of ADGRG6 downregulation.

ADGRG6 is an orphan member of the adhesion GPCR subfamily essential in the peripheral nervous system, but without any functional evidence in the mammalian olfactory system.^138^^,^^139^ Previous studies reported that ADGRG6 is expressed in both Sus cells and MVCs,^99^ and can be activated by external factors such as viruses, irritants, and bacteria (Figure 4A).

**Figure 4:**
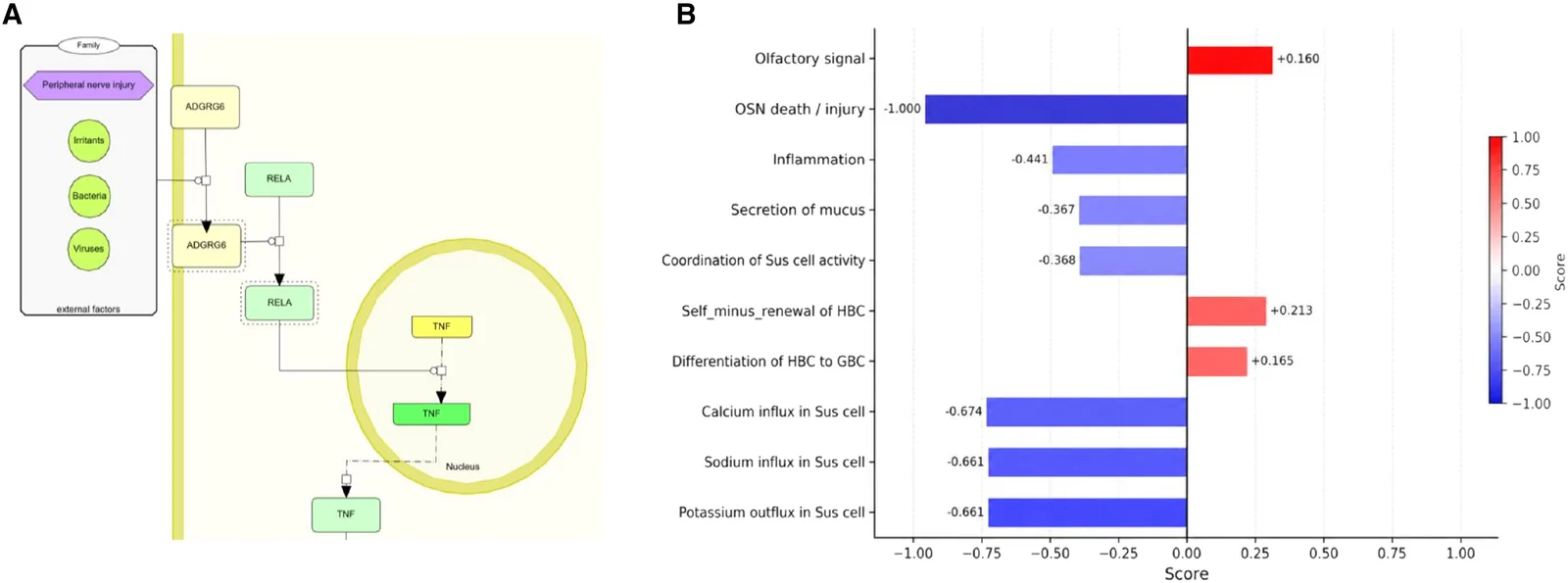
ADGRG6-RELA signaling and LUMINAR perturbation analysis in the NOSE-OE network (A) Detailed zoom-in of the molecular topology of the ADGRG6-RELA axis within the NOSE-OE network. (B) Quantitative results from the LUMINAR perturbation analysis showing the most significantly affected biological processes after *in silico* downregulation of ADGRG6. Phenotypes are colored red to indicate activation or an increase in the process (e.g., OSN proliferation and HBC self-renewal) and in blue to indicate inhibition or decreased function; scores are normalized between −1 and 1 (see STAR Methods). A step-by-step video tutorial demonstrating how to navigate and interrogate the interactive NOSE-OE map in MINERVA is provided as supplemental information (Video S1).

The LUMINAR analysis revealed that its downregulation profoundly impacts multiple signaling cascades in the Sus cells, underscoring the role of this receptor as a primary gatekeeper of epithelial homeostasis. Specifically, in our simulation, disruption of ADGRG6 expression affected 56 phenotypes within the OE, generating a quantitative map of the downstream consequences of molecular perturbations. The most affected phenotypes are shown in Figure 4B. This analysis links ADGRG6 to Sus cell excitability, including decreased Ca^2+^ influx (score −1.000, *p* = 0.009) and altered potassium efflux and sodium influx (scores −0.982, *p* = 0.009). Reduced intracellular calcium is predicted to impair activation of the transcription factor RELA (NF-κB p65), leading to altered gene expression in Sus cells. These alterations are predicted to disrupt the regulatory influence of Sus cells over growth factors, neurogenesis, and stem cell dynamics, consistent with the predicted effects on the proliferation of OSNs (score 0.972, *p* = 0.009) and the self-renewal of HBCs (score 0.339, *p* = 0.023). As Sus cells also contribute to the ionic composition of the olfactory mucus, these perturbations may alter the electrochemical gradient required for odorant detection, reflected in the observed change in olfactory signaling (score 0.267, *p* = 0.009). Overall, this analysis suggests that ADGRG6 might play a key role in regeneration and odor transduction, and its absence may trigger compensatory survival signaling or a shift in the inflammatory state of the OE, potentially linked to the RELA interaction discussed previously. These computational findings are highlighted within the NOSE-OE platform’s visual interface, enabling the tracing of the pathways behind these effects. Upon running a perturbation analysis, the platform dynamically recolors the phenotypes and biological processes across all submaps: elements are shifted toward red to indicate a positive activation or enhancement in response to the perturbation and toward blue to signify inhibition or downregulation (Video S1).

Video S1. Perturbation analysis of the NOSE-OE molecular map using MINERVA and LUMINAR, related to Figure 4 Step-by-step tutorial demonstrating navigation of the interactive NOSE-OE map and implementation of the LUMINAR perturbation analysis workflow

This distributed response pattern, which was identified in association with the ADGRG6 perturbation, shows that targeted downregulation in Sus cells leads to coordinated effects across multiple OE compartments. Frequent downstream effects on basal stem cell proliferation, mucus composition, and olfactory signal transduction resulting from perturbations affecting Sus cell signaling reinforce the central integrative role of Sus cells inferred from network topology (Figure 3). While the individual molecular components and direct interactions of this axis are fully substantiated by literature data within the OE, the functional tissue-wide outcome of this cascade remains to be experimentally verified. Users should, therefore, interpret this pathway as a validated roadmap designed to guide future validation studies.

By linking network architecture to target selection, the NOSE-OE map provides a quantitative framework for identifying molecular entry points whose modulation is predicted to produce coordinated, system-level responses, supporting translational exploration of therapeutic strategies targeting regeneration, inflammation, and sensory function. Beyond the activity propagation demonstrated here, the LUMINAR framework supports a comprehensive suite of analytical approaches, ranging from data-independent network topology evaluations to data-informed simulations that integrate multi-omics layers, baseline transcriptomic profiles, or disease-specific differential expression datasets.^43^

Signaling within the OE presents substantial challenges in neurobiology due to cellular heterogeneity, continuous and tightly regulated neurogenic turnover, and vulnerability to diverse insults including pollution, aging, head trauma, and viral infections. The NOSE-OE molecular map addresses this complexity by providing a system-level framework that integrates hundreds of molecular interactions across the cellular and compartmental organization of the OE. This atlas captures three core functional domains: odor transduction, epithelial regeneration, and inflammation, within a unified representation, which enables an integrated view of the OE function. By visualizing molecular interactions across cell types and processes, NOSE-OE supports investigation of the regulatory mechanisms underlying the OE homeostasis, sensory function, and regeneration, facilitating hypothesis generation and the identification of therapeutic targets, thanks also to the link with external datasets. Beyond single-target perturbation study, the NOSE-OE map enables systematic evaluation of how molecular perturbations affect functional processes across the OE. Perturbation analyses demonstrate that regulatory effects are not confined to individual cell types but propagate across multiple compartments, demonstrating how olfactory epithelial function arises from distributed, systems-level coordination rather than linear signaling cascades within individual cell types. The NOSE-OE atlas, therefore, captures functional crosstalk as an emergent systems property, providing a mechanistic framework to explore how local molecular perturbations propagate across compartments to influence global tissue behavior. Ultimately, by systematically linking molecular interactions to cellular and tissue-level processes, this tool provides a structured basis for investigating the mechanisms underlying olfactory functions and for identifying therapeutic targets, including modulators of signaling pathways, nasal gene delivery strategies, and long-term cell-based approaches to restore chemosensory health.

### Limitations of the study

NOSE-OE reflects the current state of experimentally supported knowledge and is, therefore, inherently dependent on the availability and quality of published evidence. The underlying studies span different experimental systems, species, cell types, and conditions, which may introduce variability in the reported molecular interactions and limit their direct comparability. Moreover, despite extensive literature curation, the signaling network cannot be considered exhaustive, as olfactory epithelial biology continues to evolve and some regulatory mechanisms remain insufficiently characterized. Finally, NOSE-OE represents a curated mechanistic knowledge framework rather than a quantitative or context-specific model; consequently, individual interactions should be interpreted in relation to their original experimental context. Future incorporation of emerging experimental evidence and complementary molecular datasets will progressively expand and refine the resource.

## Resource availability

### Lead contact

Requests for further information and resources should be directed to and will be fulfilled by the lead contact, Antonella Di Pizio (a.dipizio.leibniz-lsb@tum.de).

### Materials availability

This study did not generate new unique reagents or biological materials.

### Data and code availability

• The NOSE-OE molecular map is hosted on the MINERVA platform and is publicly accessible at https://air.elixir-luxembourg.org/minerva/index.html?id=NOSE_OE_v01.
• This paper does not report original code. Computational perturbation analyses were conducted using the LUMINAR framework, the algorithmic specifications and theoretical foundations follow.^140^
• A step-by-step tutorial demonstrating platform navigation and the perturbation workflow is provided in the supplemental information (Video S1). Any additional information required to reanalyze the data reported in this paper is available from the lead contact upon request.

## Acknowledgments

Research in the laboratory of A.D.P. is supported by the Leibniz Program for Women Professors (grant no. P116/2020). F.G. was supported by the 10.13039/100000002National Institutes of Health 10.13039/100000055National Institute on Deafness and Other Communication Disorders (grant R21DC018358) and by the Morley R. Kare Fellowship (Monell Chemical Senses Center). A.N., F.F., and A.D.P. are members of the GPCRforum (https://www.gpcrforum.org/) and FLAVOURsome COST Action (CA22161). M.V.W received support by from the German Research Foundation (DFG) for the project SYLOBIO (GRK 2901/1). S.G. and O.W. acknowledge COST Action EU-RESOLVE (EU-Resolution Biology Network, CA24138), supported by COST (European Cooperation in Science and Technology), and from the European Union’s Horizon Europe research and innovation programme through HARPNET (Marie Skłodowska-Curie Actions, Grant Agreement No. 101311747) and MICRO-NEST (Grant Agreement No. 101290494).

## Author contributions

Conceptualization, S.G., F.G., O.W., and A.D.P.; methodology, S.G., F.G., D.F., F.F., S.S.G., and M.V.W.; investigation, S.G., F.G., F.F., S.S.G., and M.V.W.; data curation, S.G., F.G., F.F., K.P.S., S.S.G., M.V.W., and M.M.K.-G.; formal analysis, S.G., F.G., and A.D.P.; software, S.G. and M.V.W.; visualization, S.G., F.G., A.D.P., F.F., and A.N.; writing – original draft, S.G., F.G., and A.D.P.; writing – review and editing, all authors; supervision, S.G., F.G., and A.D.P.; project administration, S.G., F.G., and A.D.P.; funding acquisition, F.G. and A.D.P.

## Declaration of interests

The authors declare no competing interests.

## STAR★Methods

### Key resources table

**Table:** 

REAGENT or RESOURCE	SOURCE	IDENTIFIER
**Deposited data**		

NOSE-OE interactive signaling map	This paper	https://air.elixir-luxembourg.org/minerva/index.html?id=NOSE_OE_v01

**Software and algorithms**		

MINERVA	Gawron et al.^44^	Version 21; https://minerva.pages.uni.lu/
CellDesigner	Funahashi et al.^141^	Version 4.4.2; https://www.celldesigner.org/

### Method details

#### NOSE-OE map construction

The construction of the NOSE-OE map follows the established Disease Map approach, as previously implemented in large-scale community resources such as the Atlas of Inflammation Resolution (AIR),^137^ Sarcopenia^47^ and the COVID-19 disease Map.^46^ At the core of this framework is the representation of biological knowledge using SBML and SBGN, implemented through the CellDesigner platform (http://www.celldesigner.org).^141^ Molecular entities (genes, proteins, metabolites, ions, and complexes) are represented as SBML species, while biochemical reactions, signaling events, transport processes, and regulatory interactions are encoded as directed reactions. Higher-level biological functions and experimentally observable outcomes (e.g., “olfactory signal transduction,” “mucus secretion,” or “neuroinflammatory response”) are represented as dedicated phenotype nodes, allowing downstream computational enrichment and causal analysis.

Molecular interactions and pathway topologies of NOSE-OE were reconstructed through manual expert curation of published peer-reviewed literature. More than 300 publications were systematically screened to identify experimentally validated molecular interactions relevant to OE physiology, regeneration, defense, and inflammation. Given the scope and heterogeneity of available studies, evidence was synthesized across mammalian systems, relying primarily on well-characterized rodent models for core intracellular signaling cascades while incorporating human-specific data whenever available. Manual curation criteria prioritized direct mechanistic evidence within the olfactory epithelium. Each interaction was curated with explicit directionality, biological effect (activation, inhibition, translocation, degradation), and contextual relevance. Where possible, molecular entities were annotated using stable identifiers from public databases (e.g., UniProt, HGNC), enabling interoperability with external resources and downstream data mapping. Proteins were annotated using HGNC identifiers, phenotypes were aligned to the Gene Ontology, and all molecular interactions were linked to their source evidence via PubMed identifiers, ensuring traceability and rapid content validation.

Consistent with the AIR design principles, the NOSE-OE map is organized as a modular, compartment-resolved network. Individual SBML submaps were curated to represent distinct OE cell types and functional units, including OSNs, Sus cells, MVCs, GBCs and HBC, Bowman’s glands, mucus layer, and trigeminal nerve fibers. Each submap encodes cell-type-specific molecular processes while preserving explicit spatial and functional context. Compartments were used to represent cellular and subcellular localization, enabling the differentiation between intracellular signaling, membrane-associated processes, extracellular mediators, and intercellular communication. Extracellular species act as explicit connectors between compartments, thereby enabling the systematic representation of paracrine signaling, neuroimmune interactions, and epithelial–neuronal crosstalk.

#### Integration into the MINERVA platform

Following curation, all SBML submaps were integrated into the MINERVA platform, a web-based environment specifically designed for hosting, visualizing, and analyzing Disease Maps.^44^ MINERVA enables interactive navigation across map layers, database-linked annotations, and dynamic visualization of molecular states.

The NOSE-OE MINERVA instance supports: 1) hierarchical exploration from phenotype-level processes to detailed molecular mechanisms, 2) cross-linking of shared molecular components across submaps, and 3) extensibility for future incorporation of regulatory layers (e.g., transcriptional, post-transcriptional, or lipid mediator signaling). In addition to interactive visualization, the NOSE-OE MINERVA instance is linked to the LUMINAR (Leveraging Unified Molecular Interaction Networks for Analysis and Reasoning) plugin suite, which is accessible directly through the platform and broadens the accessibility to the maps.

#### Network-based perturbation propagation and phenotype scoring

Perturbation propagation analyses were conducted using the current release of the LUMINAR framework, whose underlying parameters, algorithmic settings, and theoretical foundation are described in detail in.^140^ To ensure complete transparency and independent reproducibility, step-by-step instructions and parameter selections for reproducing the perturbation workflows reported in this study are demonstrated in the accompanying video tutorial (Video S1, Perturbation analysis of the NOSE-OE molecular map using MINERVA and LUMINAR, related to Figure 4).

We represent the NOSE-OE network as a directed graph G=(V,E), where V denotes the set of molecular entities and phenotype nodes, and E⊆V×V denotes directed regulatory interactions. Each edge $e_{ij}\in E$ is assigned a sign $s_{ij}\in \left\{+1,-1\right\}$, corresponding to activating or inhibitory effects, respectively, as curated from experimental literature. A molecular perturbation, such as downregulation or overexpression of a target node $v_{k}$, is represented as an initial perturbation vector p, where $p_{k}\neq 0$ and all other entries are set to zero. The propagation of this perturbation through the network reflects the cumulative influence of upstream and downstream regulatory paths and interaction signs, consistent with the previously validated AIR workflow.^140^

For each phenotype node φ∈V, an aggregate influence score is computed by integrating all directed paths connecting the perturbed node to φ, weighted by interaction sign and normalized by network topology:

$$S\left(\varphi \right)=\sum_{i\in U\left(\varphi \right)}\;p_{i}\cdot s_{i\varphi }$$

where U(φ) denotes the set of upstream regulators of phenotype φ. The resulting phenotype scores are normalized to the interval [−1,+1], enabling direct comparison across perturbations and phenotypes. Statistical significance of phenotype perturbation scores is assessed using permutation-based background distributions, yielding associated *p*-values.^140^ This framework allows quantitative assessment of how localized molecular perturbations propagate across compartments and manifest as system-level functional changes.

## Footnotes

Supplemental information can be found online at https://doi.org/10.1016/j.isci.2026.117818.
